# Disrupted Rhythms, Disrupted Microbes: A Systematic Review of Shift Work and Gut Microbiota Alterations

**DOI:** 10.3390/nu17172894

**Published:** 2025-09-07

**Authors:** Diego Grasa-Ciria, Sergio Couto, Eva Samatán, Begoña Martínez-Jarreta, María del Carmen Cenit, Isabel Iguacel

**Affiliations:** 1 Hospital Universitario Miguel Servet, 50009 Zaragoza, Spain; diegograsa0@gmail.com (D.G.-C.); iguacel@unizar.es (I.I.); 2 Faculty of Health Sciences, University of Zaragoza, 50009 Zaragoza, Spain; 3 Hospital Clínico Universitario Lozano Blesa, 50009 Zaragoza, Spain; eva.m.samatan@gmail.com; 4 Scientific Research Group GIIS-063 (IIS-Aragón), Occupational Medicine, Faculty of Medicine, University of Zaragoza, 50009 Zaragoza, Spain; mjarreta@unizar.es; 5 Instituto Universitario de Investigación en Ciencias Ambientales de Aragón (IUCA), University of Zaragoza, 50009 Zaragoza, Spain; 6 Department of Nutrition and Sustainable Animal Production, Estación Experimental del Zaidín (EEZ-CSIC), 18008 Granada, Spain; mcarmen.cenit@eez.csic.es; 7 Centro de Investigación Biomédica en Red de Fisiopatología de la Obesidad y Nutrición, 28029 Madrid, Spain; 8 Instituto de Investigación Sanitaria Aragón, 50009 Zaragoza, Spain; 9 Instituto Agroalimentario de Aragón, 50013 Zaragoza, Spain

**Keywords:** shift work, gut microbiota, gut microbiome, chronodisruption, circadian rhythm, dysbiosis, intestinal microbiota

## Abstract

**Background**: Shift work, especially during nighttime hours, disrupts the circadian system and is linked to higher rates of metabolic, gastrointestinal, cardiovascular, and neurocognitive disorders. Emerging evidence suggests that gut microbiota may mediate these associations. This systematic review assessed whether shift work alters gut microbiota composition and explored potential health consequences. **Methods**: A systematic search was conducted in PubMed, Scopus, and ScienceDirect from inception to March 2025. Studies reporting gut microbiota alterations in adult shift workers were included. Two reviewers independently screened articles and extracted data. Risk of bias was assessed using the NIH Quality Assessment Tool and the ROBINS-E framework. Five studies met the eligibility criteria and were included in the final synthesis. **Results**: The selected studies comprised four observational investigations with small sample sizes and one Mendelian randomization study leveraging large-scale genetic datasets. Observational studies reported reduced α-diversity and increased relative abundance of pro-inflammatory genera—including *Escherichia*/*Shigella*, *Blautia*, and *Dialister*—in night shift workers. These microbiota alterations were associated with gastrointestinal complaints and indicators of cardiometabolic dysfunction. The Mendelian randomization study provided preliminary evidence supporting a causal relationship between circadian misalignment, gut dysbiosis, and increased cardiovascular risk. **Conclusions**: Shift work is associated with significant alterations in gut microbiota composition that may contribute to adverse health outcomes. However, current evidence is limited and heterogeneous, preventing firm causal conclusions. Further high-quality longitudinal and interventional research is needed to clarify underlying mechanisms and inform preventive strategies.

## 1. Introduction

The expansion of global economies, the rise of 24/7 services, and the acceleration of technological advancement have profoundly reshaped labor dynamics in recent decades. One prominent result is the widespread adoption of shift work, particularly in essential sectors such as healthcare, transportation, manufacturing, and public safety, where continuous coverage is imperative [1]. It is estimated that up to 20% of the working EU population in industrialized countries is engaged in shift schedules that include night work, early morning shifts, or rotating patterns. Although these schedules fulfill operational needs, they are increasingly recognized for their substantial physiological costs.

Shift work disrupts circadian rhythms, which are regulated by central and peripheral biological clocks in coordination with environmental cues such as light exposure and feeding times. Circadian rhythms, governed by the suprachiasmatic nucleus and peripheral clocks, play a critical role in regulating sleep–wake cycles, endocrine rhythms, immune responses, gastrointestinal function, and metabolic homeostasis [2,3]. Disruption of these rhythms, known as circadian misalignment, can lead to a cascade of biological dysregulations, including sleep disturbances, increased sympathetic activity, altered glucose and lipid metabolism, systemic inflammation, and changes in cardiovascular tone [4,5,6]. Over time, this misalignment has been associated with a heightened risk of chronic non-communicable diseases, including obesity, type 2 diabetes, cardiovascular disease, gastrointestinal disorders, mood disorders, and certain types of cancer [7,8,9,10].

Due to these health risks, international organizations such as the World Health Organization (WHO) and the International Agency for Research on Cancer (IARC) have classified night shift work as a probable human carcinogen and issued guidelines to protect exposed workers. Nevertheless, despite clear epidemiological associations, the biological mechanisms underlying these health effects remain only partially understood, hindering the development of targeted prevention strategies [11].

Recent research has focused on the gut microbiota as a potential mediator linking circadian disruption to adverse health outcomes. The gut microbiota comprises trillions of microorganisms—including bacteria, viruses, fungi, and protozoa—that inhabit the gastrointestinal tract and have co-evolved with the host over time [12]. It plays a fundamental role in maintaining host physiological homeostasis by influencing digestion, nutrient absorption, metabolic regulation, immune function, and neuroendocrine signaling, particularly through the gut–brain axis [13,14].

Importantly, the gut microbiota is not static but exhibits circadian fluctuations in both composition and function. These microbial rhythms are driven by host-related cues such as feeding schedules, hormonal cycles (e.g., cortisol, melatonin), and even locomotor activity, all of which are governed by the host’s circadian clocks [15,16,17]. When these cues are altered by shift work—through sleep deprivation, irregular eating schedules, or nocturnal light exposure—the temporal organization of the microbiota can become desynchronized, leading to dysbiosis [18,19].

Dysbiosis refers to an imbalance in gut microbial composition and function, characterized by reduced microbial diversity, an increased abundance of pro-inflammatory taxa such as *Escherichia*/*Shigella*, *Blautia*, and *Dialister*, and a decreased presence of beneficial, anti-inflammatory bacteria [20,21,22]. These alterations have been associated with increased intestinal permeability, low-grade systemic inflammation, insulin resistance, and dysregulated lipid metabolism all hallmarks of metabolic syndrome and other related chronic conditions [23,24].

Animal studies have shown that circadian misalignment alone can shift gut microbiota composition and disrupt metabolic and immune functions; however, these are all preclinical studies, making it difficult to emphasize their experimental nature [17,19]. Similar patterns have been observed in small human studies of shift workers, particularly in healthcare settings, where microbial alterations have been associated with functional gastrointestinal symptoms, cognitive impairments, and early indicators of cardiometabolic risk [25,26,27,28,29,30].

Nevertheless, emerging research suggests that gut microbiota profiles may serve as early biomarkers of susceptibility to shift work–related disorders and represent promising targets for therapeutic interventions [31,32,33,34,35]. In this regard, strategies such as chrononutrition and time-restricted feeding [31,33], circadian-based modulation of the gut microbiome [32], probiotic supplementation [34], and light-based interventions to counteract circadian misalignment [35] are being investigated for their potential to restore both microbial and circadian homeostasis, although definitive clinical evidence in shift workers is still lacking. These approaches may help mitigate the adverse health effects of circadian disruption, particularly among individuals chronically exposed to irregular work schedules.

Given the increasing prevalence of shift work and its significant health implications, there is an urgent need for a deeper understanding of the interplay between shift schedules, circadian disruption, and the gut microbiota. This systematic review aims to critically synthesize human evidence linking shift work to changes in gut microbiota composition, to identify consistent microbial signatures, evaluate physiological and clinical consequences, and highlight methodological gaps and future research directions.

## 2. Materials and Methods

This systematic review was conducted to examine the relationship between shift work and gut microbiota alterations by analyzing all relevant studies published up to 2025. A comprehensive and rigorous process for the literature search, study selection, and critical appraisal was carried out in accordance with the PRISMA (Preferred Reporting Items for Systematic Reviews and Meta-Analyses) guidelines to ensure methodological transparency and reproducibility (Supplementary Material S1).

The primary objective was to synthesize current scientific evidence on how shift work and the resulting circadian rhythm disruption affect gut microbiota composition and function, as well as to explore the associated metabolic, immune, and neurocognitive implications. Detailed search strategies, selection procedures, and methods for data extraction and synthesis are described below.

### 2.1. Study Design

This study is a systematic review conducted in accordance with PRISMA guidelines and registered in the PROSPERO database (registration number: CRD420251018697) [36,37].

### 2.2. Search Strategy

The literature search was conducted between February and March 2025 using three major scientific databases: PubMed, Scopus, and ScienceDirect, selected for their comprehensive coverage of health sciences, microbiology, and chronobiology. ScienceDirect was included as a complementary database. Although its coverage focuses primarily on journals published by Elsevier and may be considered more limited than other databases, it hosts a considerable number of high-impact journals in microbiology, physiology, and health sciences. Its inclusion allowed us to broaden the search and minimize the risk of omitting relevant studies, in line with methodological recommendations for comprehensive systematic reviews.

A combination of English terms with Boolean operators was used, including keywords and MeSH terms such as: “microbiota,” “gut microbiota,” “dysbiosis,” “shift work,” “night shift,” “circadian disruption,” “sleep deprivation,” “rotating shifts,” “intestinal microbiome,” and “sleep quality” (Supplementary Material S2).

#### Search Strategy Justification

Different search terms were applied across databases to account for variations in indexing systems, search fields, and controlled vocabularies. PubMed allows the use of MeSH terms and title/abstract searches, while Scopus and ScienceDirect rely primarily on keyword and title/abstract searches. To maximize sensitivity and ensure comprehensive coverage, the search strategy was adapted for each database while preserving the same conceptual framework. This approach ensured that relevant studies were captured despite differences in database architecture, maintaining consistency in the evaluation of shift work and gut microbiota relationships.

### 2.3. Eligibility Criteria

To clearly define the research question and guide the systematic search and study selection, the PICO (Population, Intervention, Comparison, Outcome) framework proposed by Richardson et al. (1995) was used [38]. This framework ensures a structured identification of relevant evidence and methodological consistency throughout the review. The PICO components were tailored to focus specifically on adult human populations exposed to shift work, with particular attention to gut microbiota changes. Table 1 summarizes the specific PICO elements applied in this review, which directly informed the inclusion and exclusion criteria described below.

Inclusion Criteria: We included original, peer-reviewed studies that investigated alterations in the human gut microbiota associated with shift work, particularly night or rotating schedules. Eligible studies were required to involve adult human participants and to perform microbiota analyses based on stool samples, using either culture-independent methods or genomic sequencing techniques. Both observational and interventional study designs were considered, including cross-sectional studies, cohort studies, pilot investigations, and Mendelian randomization analyses. Only articles published in English or Spanish were included.

Exclusion Criteria: Studies were excluded if they involved animal models, in vitro experiments, or populations not exposed to shift work. Reviews, editorials, conference abstracts, and case reports were also excluded. Additionally, studies that focused exclusively on sleep disorders or circadian disruption without specific reference to shift work exposure were not considered eligible. Research lacking original data on gut microbiota composition or functionality was likewise excluded from the analysis.

### 2.4. Study Selection Process

**Figure 1:**
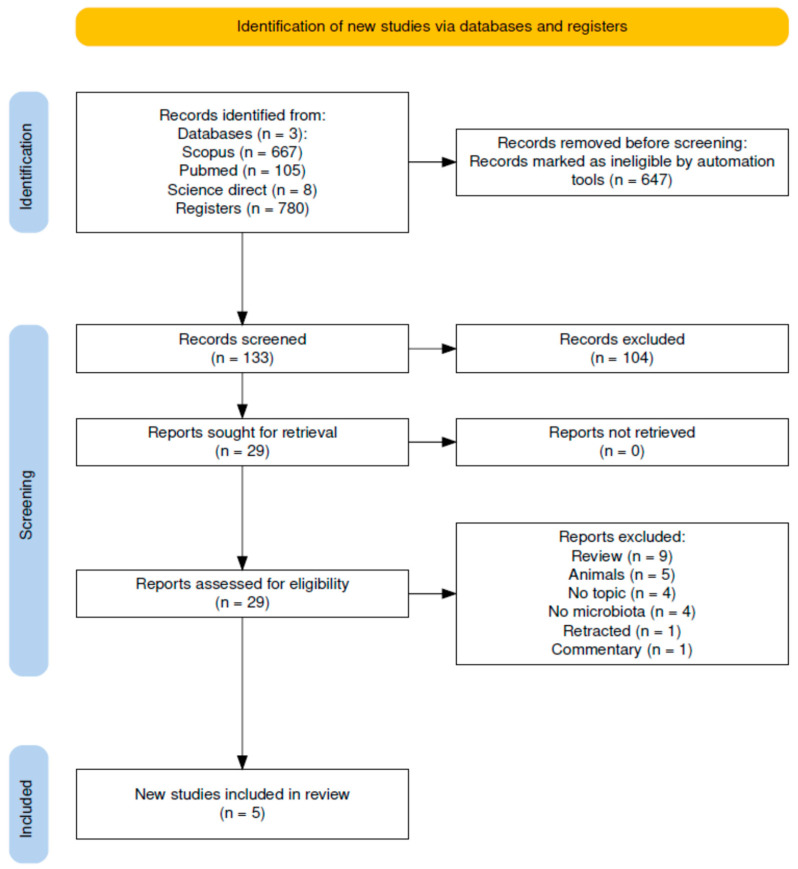
Flow diagram of the literature search process.

A total of 780 articles were initially identified through the systematic database search. After removing duplicates, two independent reviewers (DG and SC) screened the titles and abstracts according to the predefined inclusion and exclusion criteria. Studies that clearly failed to meet the eligibility criteria were excluded at this stage. The full texts of the remaining articles were then retrieved and evaluated in depth. This evaluation considered methodological quality, the clarity and relevance of the findings, and their alignment with the review objective. Any discrepancies between reviewers were resolved through discussion and, when necessary, by consultation with a third reviewer. The complete selection process is illustrated in the PRISMA flow diagram (Figure 1).

### 2.5. Data Extraction

For each included study, relevant data were systematically extracted using a standardized data collection template. Extracted variables included study design, sample size, population characteristics, type and duration of shift work exposure, microbiota assessment methods (e.g., 16S rRNA sequencing), main findings related to microbiota composition and diversity, reported clinical or physiological outcomes (e.g., gastrointestinal, metabolic, or cardiovascular effects), and key methodological limitations. This structured approach enabled the identification of core patterns and associations across studies, forming the basis for qualitative synthesis.

### 2.6. Data Synthesis and Analysis

The data extracted from the selected studies were analyzed qualitatively. A descriptive comparison of findings across studies was performed to identify similarities, discrepancies, and recurring patterns regarding the relationship between shift work, circadian disruption, and gut microbiota. This narrative synthesis enabled the integration and comparison of findings across studies with diverse methodological designs, providing a more comprehensive and nuanced understanding of the phenomenon under investigation.

### 2.7. Assessment of Methodological Quality and Risk of Bias

The methodological quality and risk of bias of the included studies were assessed using two complementary tools: the ROBINS-E (Risk Of Bias In Non-randomized Studies—of Exposures) tool [39] and the NIH Quality Assessment Tool [40] (Supplementary Materials S3 and S4). These tools are well-suited for evaluating observational studies with naturally occurring exposures such as shift work. Their combined use ensured a thorough and context-appropriate assessment across different study designs, providing greater confidence in the validity of our findings.

ROBINS-E was applied to all five studies to evaluate bias across seven domains related to confounding, selection of participants, exposure classification, deviations from intended exposures, missing data, outcome measurement, and selection of reported results. Each domain was rated as low, moderate, or serious risk of bias. Additionally, the NIH Quality Assessment Tool for Observational Cohort and Cross-Sectional Studies (14-item version) was applied uniformly across all studies, regardless of specific design, to ensure a consistent appraisal of methodological quality.

All assessments were conducted independently by two reviewers, with discrepancies resolved through consensus or by consulting a third reviewer. This dual-review process ensured a rigorous and transparent appraisal of methodological quality and potential bias, enhancing the reliability and interpretability of the findings presented in this systematic review.

## 3. Results

### 3.1. Search and Study Selection

A total of 780 articles were identified through the search strategy. After removing duplicates, two reviewers independently screened titles and abstracts to exclude non-relevant studies. The remaining full-text articles were thoroughly assessed. Ultimately, five studies that met the predefined inclusion criteria were analyzed. This process was conducted in accordance with PRISMA guidelines [36] and is illustrated in Supplementary Material S1.

### 3.2. Methodological Quality Assessment and Risk of Bias Assessment

This systematic review highlights a limited and heterogeneous body of evidence, characterized by several methodological shortcomings. The methodological quality assessment, conducted using the NIH Quality Assessment Tool, indicates that while some studies demonstrate robust design, appropriate sample selection, and adequate group comparison, others are limited by small sample sizes, insufficient control for potential confounders (e.g., lifestyle, stress, dietary habits), and lack of clarity in participant selection procedures [40].

The risk of bias assessment using the ROBINS-E tool revealed moderate to high risk across several key domains, particularly regarding participant selection, exposure classification, control of confounding variables, and outcome measurement [39] (Supplementary Materials S3 and S4). The absence of a standardized definition of “shift work,” combined with methodological limitations and inadequate adjustment for confounders, reduces both the comparability across studies and the internal validity of their findings.

Overall, while current evidence suggests a potential association between shift work and alterations in gut microbiota, methodological weaknesses and risk of bias limit the strength and generalizability of these findings. Future research should aim for larger sample sizes, standardized definitions of shift work, and more rigorous control of confounding factors to improve study validity and reliability.

### 3.3. Characteristics of Included Studies

A total of five studies met the inclusion criteria for this systematic review, encompassing diverse methodological approaches. None were randomized controlled trials. Four studies employed observational designs, while one study applied a Mendelian randomization framework to examine potential causal relationships. Most cohorts were small, ranging from 10 to 51 participants, except for one large genetic study by Zhang et al., 2024 [41], which analyzed data from over 263,000 individuals. This heterogeneity in study design and sample size reflects the emerging nature of this research field and highlights the need for both mechanistic studies and large-scale epidemiological investigations.

Geographically, two studies were conducted in Turkey (Mortaş et al., 2020 and 2022) [25,27], one in the United States (Rogers et al., 2021) [26], one in Taiwan (Yao et al., 2025) [42], and one used UK Biobank data (Zhang et al., 2024) [41]. The populations studied were heterogeneous, predominantly composed of healthcare professionals and individuals engaged in night or rotational shift work. Specifically, Mortaş et al., 2020 [27] recruited 10 male security officers aged 25–40 years working rotating shifts and evaluated compositional changes in the gut microbiota between day and night shifts. The same cohort was later used in a follow-up study by Mortaş et al., 2022 [25], which focused on functional outcomes, including short-chain fatty acid (SCFA) profiles and gut barrier integrity. Rogers et al., 2021 [26] studied 51 full-time nurses aged 21–59 years working 12 h shifts, while Yao et al., 2025 [42] included 15 healthcare professionals exposed to at least four consecutive night shifts followed by a three-day recovery period. Lastly, Zhang et al., 2024 [41] conducted a two-sample Mendelian randomization study using genetic and lifestyle data from a large and ethnically heterogeneous adult population, including over 263,000 individuals, predominantly of European ancestry

Microbiota analyses varied among studies and included assessments of alpha and beta diversity, taxonomic composition at various phylogenetic levels, and, in some cases, predicted microbial functions using bioinformatic tools. Most studies employed 16S rRNA gene sequencing, although the specific sequencing platforms and bioinformatic pipelines were not consistently reported. Observed outcomes included reductions in microbial diversity, increased relative abundance of pro-inflammatory taxa such as Escherichia/Shigella, Blautia, Dialister, and Gemellaceae, as well as associations with gastrointestinal symptoms (e.g., abdominal pain, bloating, altered bowel habits), metabolic parameters (glucose and lipid profiles), and cardiovascular risk markers. However, due to differences in sample size, exposure duration, occupational context, and analytical techniques, definitive conclusions remain limited.

In summary, the heterogeneity in study designs, population characteristics, shift work exposures, and microbiota profiling methods underscores the complexity of investigating gut microbiota alterations in the context of circadian disruption. While this variability poses challenges for meta-analytical synthesis, it provides a broad and multi-dimensional perspective on potential interactions between shift work and host–microbiome dynamics across different occupational and cultural settings.

### 3.4. Synthesis of Results

The studies included in this review consistently indicate that exposure to shift work, particularly during nighttime hours, is associated with measurable alterations in gut microbiota diversity and taxonomic composition. However, the magnitude and specific nature of these alterations varied across studies, depending on population characteristics, duration of shift work exposure, and methodological approaches.

For example, Mortaş et al., 2020 [27] evaluated the effects of rotational shift work on gut microbiota composition, comparing day versus night shifts in 10 male security officers. Their results revealed shifts in the relative abundance of specific bacterial taxa, including a reduction in *Bacteroidetes* and an increase in *Firmicutes* and *Actinobacteria*, with Dorea longicatena and Dorea formicigenerans being more abundant during night shifts. This study highlighted how circadian misalignment induced by shift work can alter microbial composition and potentially contribute to metabolic and gastrointestinal risk.

In contrast, Mortaş et al., 2022 [25], using the same cohort but focusing on intestinal function and metabolite profiles, assessed short-chain fatty acids (SCFAs) and gut barrier integrity. They found that SCFA concentrations (acetic, propionic, and total SCFA) were associated with intestinal permeability markers during night shifts, and that dietary patterns modulated these effects. This study emphasized the functional consequences of circadian disruption on gut microbiota–host interactions, rather than compositional changes per se.

Rogers et al., 2021 [26] found no overall differences in microbial diversity between day and night shift workers. Nevertheless, intra-individual analyses revealed significant shifts in alpha and beta diversity across shifts, with alpha diversity increasing after day shifts and decreasing after night shifts. Seven Amplicon Sequence Variants (ASVs) showed significant changes over time, three of which were associated with irritable bowel syndrome (IBS) symptoms.

The pilot study by Yao et al., 2025 [42] analyzed healthcare workers recently exposed to night shifts and assessed both gut microbiota and brain connectivity. Although no global shifts in microbial diversity were observed, specific alterations in the Gemellaceae family were identified, correlating with changes in functional brain network activity.

Finally, the Mendelian randomization study by Zhang et al., 2024 [41] examined potential causal links between shift work, gut microbiota alterations, and cardiometabolic risk. Their analysis revealed significant genetic associations linking night shift exposure with specific changes in gut microbial composition, alongside an elevated risk of hypertension and ischemic heart disease.

Collectively, the reviewed studies support the hypothesis that shift work, particularly night shifts, disrupts gut microbial homeostasis. However, notable heterogeneity exists across studies in terms of design, population characteristics, and methodological approaches. Study sample sizes range from small pilot cohorts to large-scale genetic datasets, and gender composition varies—some studies focused exclusively on female nurses, whereas others included mixed-gender occupational groups.

Countries of origin and age ranges also vary considerably, potentially influencing microbiota composition through geographic, dietary, and lifestyle factors. Moreover, the type and duration of shift work exposure are inconsistently reported, further complicating cross-study comparisons. These methodological and demographic discrepancies limit the generalizability of the findings and highlight why microbial alterations may differ in magnitude and clinical relevance depending on the severity, chronicity, and context of circadian disruption (Table 2).

Table 3 provides an integrated overview of the main clinical outcomes associated with gut microbiota alterations reported in the included studies. It summarizes key observations from each study, contextualized through structured quality assessments and domain-based evaluations of risk of bias. This synthesis highlights the heterogeneity of clinical manifestations—ranging from gastrointestinal symptoms and metabolic alterations to neurocognitive changes—as well as the varying methodological rigor across studies. By incorporating aspects such as study design, sample representativeness, and analytical consistency, the table allows a more critical interpretation of the reliability, robustness, and translational relevance of the reported findings. Study-specific limitations are also noted to inform future research directions and guide the interpretation of current evidence.

## 4. Discussion

### 4.1. Summary of Main Findings

This systematic review aimed to determine whether shift work, particularly night shifts, alters gut microbiota composition in adults and how these changes relate to health outcomes. Despite the limited number of eligible studies, a consistent pattern emerged: shift work is associated with measurable alterations in gut microbial diversity and composition.

Several studies reported reduced α-diversity and an enrichment of pro-inflammatory taxa such as *Escherichia*/*Shigella*, *Blautia*, and *Dialister*. These microbial changes were often linked to markers of metabolic dysfunction, gastrointestinal symptoms, and low-grade systemic inflammation, suggesting that dysbiosis may mediate some of the adverse health effects of circadian misalignment [25,26,27,41,42].

### 4.2. Mechanistic Insights

Circadian disruption inherent to shift work affects both central and peripheral biological clocks, impacting gut motility, barrier integrity, immune function, and secretion cycles [43,44]. Altered melatonin and cortisol rhythms may disrupt microbial rhythmicity and impair intestinal barrier function, potentially driving systemic metabolic disturbances [43,45].

Microbial metabolites—particularly SCFAs—act as mediators by influencing epithelial integrity, peripheral clock gene expression, and host metabolic regulation [25,46]. The Mendelian randomization study by Zhang et al., 2024 [41] adds further support by suggesting a potential causal relationship between shift work, gut microbiota changes, and increased cardiometabolic risk [47].

### 4.3. Role of Diet and Behavioral Factors

Dietary and behavioral factors, such as irregular eating patterns and nocturnal food intake, may further exacerbate microbial disturbances. Mortaş et al., 2022 [25] reported that high-sugar diets amplified microbial alterations and negatively affected SCFA production, which is closely linked to gut barrier function. These findings highlight the complex interplay between circadian disruption, diet, and microbial metabolism, and suggest that nutritional interventions may help mitigate adverse effects [46,48].

### 4.4. Clinical and Occupational Implication

From a clinical and occupational health perspective, these alterations may contribute to the elevated risk of metabolic syndrome, gastrointestinal disorders, and neurocognitive or affective disturbances observed in shift-working populations. Microbial profiling could serve as a non-invasive biomarker for monitoring circadian disruption and guiding preventive strategies. Interventions such as time-restricted feeding, probiotics, or chronotype-adjusted shift schedules show promise, although robust evidence in human cohorts remains limited [31,32,33,34,35,49].

### 4.5. Strengths and Limitations of the Review

This systematic review has several strengths. First, it is the first review to synthesize evidence specifically examining the relationship between shift work and gut microbiota alterations in adult populations, addressing a novel and increasingly relevant topic in occupational and circadian health. Second, the review followed PRISMA guidelines, ensuring transparency in study selection, data extraction, and risk of bias assessment. Third, the use of two independent reviewers, along with structured tools (ROBINS-E and NIH quality assessment), provided a rigorous evaluation of methodological quality and reduced subjective bias. Finally, the inclusion of diverse study designs, including observational cohorts and Mendelian randomization analyses, offers a broader perspective on both associative and potentially causal links.

However, several limitations must also be acknowledged. The number of eligible studies was small (*n* = 5), limiting the generalizability of findings and precluding quantitative synthesis through meta-analysis. There was substantial heterogeneity across studies in terms of population characteristics, shift work schedules (e.g., duration, intensity, and schedule), sample collection timing, microbiome analysis methods, and outcome reporting. Additionally, although quality assessment tools were applied, the included studies were generally of moderate to low methodological quality, with common issues including small sample sizes, lack of dietary control, and insufficient adjustment for confounding factors such as diet, medication use, and psychosocial stress. Finally, due to the novelty of the field, most studies had short follow-up periods, limiting insights into long-term effects or adaptive processes.

Despite these limitations, the review identifies consistent patterns and offers valuable insights into the potential mechanisms linking circadian disruption to gut dysbiosis. It also provides a critical foundation for future research directions in this emerging area.

### 4.6. Research Gaps and Future Directions

Several important research gaps remain in understanding the relationship between shift work and gut microbiota alterations. The very limited number of eligible studies underscores the novelty of this research area and the current scarcity of high-quality evidence. Future studies should prioritize the use of well-designed, larger-scale, and longitudinal cohort designs to capture the temporal dynamics of microbiota changes and identify critical periods of vulnerability or adaptation. Randomized controlled trials are urgently needed to evaluate the potential therapeutic effects of microbiota-targeted interventions—such as time-restricted feeding, probiotics, and prebiotics—specifically in shift-working populations. In addition, the integration of multi-omics approaches, including metagenomics, metabolomics, and transcriptomics, will be essential to establish links between microbial composition, functional activity, and host physiology. To enhance the personalization and translational relevance of future findings, individual-level factors such as chronotype, sex, genetic background, dietary patterns, and occupational characteristics must also be systematically considered. Furthermore, it is crucial to investigate whether gut microbial communities themselves may contribute to circadian misalignment, potentially creating bidirectional feedback loops that exacerbate dysbiosis and downstream health effects [48,49]. Addressing these gaps will be vital for the development of effective, evidence-based strategies to prevent or mitigate the adverse health consequences of shift work. Advancing this field will require coordinated, interdisciplinary collaboration across occupational health, chronobiology, nutrition, and microbiome research.

## 5. Conclusions

In summary, shift work—particularly night shifts—is consistently associated with significant alterations in gut microbiota diversity and composition. This dysbiosis may act as a key mediating factor in the development of metabolic, gastrointestinal, and cardiovascular disorders, substantially increasing the health burden for shift-working populations. While the existing evidence is promising, it is constrained by methodological heterogeneity, small sample sizes, and inadequate control of confounding factors such as diet, lifestyle, psychosocial stress, and sleep quality. These limitations hinder definitive causal conclusions and limit the generalizability of findings across diverse populations. The observations reported in the five studies included in this review provide a foundational, though preliminary, understanding of these relationships.

This review highlights the gut microbiome as a central mediator of shift work–related health risks and underscores the urgent need for high-quality, longitudinal, and multidimensional studies employing rigorous, standardized methodologies, with careful consideration of individual factors such as chronotype and sex. From an occupational health perspective, incorporating microbiota assessment as a biomarker for shift work–related risk could enable earlier prevention and the development of tailored interventions.

Future research should prioritize the integration of multi-omics approaches alongside interventional trials to clarify underlying mechanisms and inform targeted preventive and therapeutic strategies. Such advances are essential to mitigate the adverse effects of shift work–induced gut microbiota alterations and ultimately safeguard the health of at-risk shift-working populations.

## Figures and Tables

**Table 1:** PICO elements.

PICO Element	Description	Inclusion Criteria	Exclusion Criteria
**Population (P)**	Adult human participants	Adults (≥18 years) exposed to shift work (night or rotating shifts)	Animal models, in vitro studies, or populations not exposed to shift work
**Intervention (I)**	Exposure to shift work	Exposure to night shifts, rotating shifts, or other work schedules involving circadian disruption	Studies that do not specify shift work as the exposure of interest
**Comparison (C)**	Day workers or non-shift workers (when applicable)	Studies that include a comparison group of non-shift workers or baseline/control data	Studies lacking a clear reference or comparison group (if applicable to the study design)
**Outcome (O)**	Changes in gut microbiota composition or function	Studies reporting gut microbiota outcomes (e.g., diversity indices, taxonomic shifts, dysbiosis), assessed via stool sample analysis or genomic sequencing	Studies without original microbiota data, or focusing exclusively on circadian disruption or sleep without reference to shift work
**Study Design**	Type of research included	Original, peer-reviewed observational or experimental studies (e.g., cross-sectional, cohort, pilot, Mendelian randomization)	Reviews, editorials, case reports, or conference abstracts

**Table 2:** Summary of findings from included studies on shift work and gut microbiota.

Study (First Author, Year)	Design	Sample/Shift Type	Shift Work Definition	Shift Work Exposure Duration	Gender	Country	Age Range
Mortaş et al., 2020 [27]	Prospective, longitudinal, observational study	10 security officers; rotational shifts	Day shift (07:00–15:00 h) for 4 weeks and after working the night shift (23:00–07:00 h) for 2 weeks.	September 2017–October 2018	10 males	Turkey	25–40 years
Mortaş et al., 2022 [25]	Prospective, longitudinal, within-subject observational study	10 security officers; rotational shifts	Day shift (07:00–15:00 h) for 4 weeks and after working the night shift (23:00–07:00 h) for 2 weeks	September 2017–October 2018	10 males	Turkey	25–40 years
Rogers et al., 2021 [26]	Prospective, longitudinal, pre-post pilot study	51 full-time nurses; 12 h day or night shifts	47% straight day shifts, 51% night shifts, 2% rotating shifts	Unspecified duration	49 females, 2 males	United States	18–65 years
Yao et al., 2025 [42]	Prospectiove, longitudinal, observational pilot study	15 healthcare workers; 4-night shifts + 3 rest days	Consecutive shift work for at least 4 days with a 3-day resting period after shifts	Unspecified duration	11 females, 4 males	Taipei, China	20–65 years
Zhang et al., 2024 [41]	Mediation, mendelian randomisation study with cohort studies	263,315 European individuals with jobs involving shift work and 18,340 individuals with microbiota information	Jobs involving shift work (JSW) from the UK Biobank	Not specified	Non-specified but heterogeneous in terms of ethnic background, age, male/female ratio	Mainly Europeans but also from Middle Eastern, East Asian; American Hispanic/Latin and African American countries.	Adults

**Table 3:** Summary of gut microbiota alterations and associated clinical outcomes in shift work: key findings from included studies.

Study	Main Microbiota Findings	Associated Clinical Outcomes	Key Observations	Key Limitations	NIH Quality Assessment	ROBINS-E Risk of Bias
Mortaş et al., 2020 [27]	*Bacteroidetes* decreased; *Actinobacteria* and *Firmicutes* increased during night shifts	Potential metabolic and inflammatory implications due to altered Firmicutes/Bacteroidetes ratio	Gut dysbiosis possibly linked to night shift exposure	Small sample, healthy individuals only	55%	High risk
Mortaş et al., 2022 [25]	Increased *Blautia*, *Bifidobacterium*, *Dialister*, *Ruminococcus gnavus* after night shifts; more pronounced with high sugar diet	Reduced short-chain fatty acid (SCFA) production potentially compromising intestinal barrier function	Microbiota changes observed independent of major dietary or biochemical shifts	Small sample, no repeated samples	58%	High risk
Rogers et al., 2021 [26]	Significant within-shift changes in alpha/beta diversity; three Amplicon Sequence Variants (ASVs), associated with irritable bowel syndrome (IBS) symptoms	IBS symptoms potentially associated with microbial diversity and ASVs changes	Significant ASVs changes detected in relation to symptom clusters	Volunteer bias, limited representativeness	60%	Some concerns
Yao et al., 2025 [42]	No global changes in diversity; *Gemellaceae* family altered, correlating with brain connectivity shifts	Altered functional brain connectivity associated with *Gemellaceae* abundance during shifts	Brain dan/dmn (default mode y dorsal attention) connectivity changed dynamically; *Gemellaceae* correlated with network activity changes	Diet not controlled; cultural context	67%	Some concerns
Zhang et al., 2024 [41]	*Eubacterium* brachy group mediates Jobs involving Shift Work (JSW) effect on hypertension and Coronary Heart Disease (CHD) risk	Elevated risk of hypertension and CHD mediated by gut microbial taxa	Eubacterium brachy identified as key mediator linking JSW to increased cardiometabolic risk	Summary-level data; self-reported exposure; genus-level resolution	90%	Some concerns

## Data Availability

The original contributions presented in this study are included in the article/Supplementary Materials. Further inquiries can be directed to the corresponding author.

## Supplementary Materials

The following supporting information can be downloaded at https://www.mdpi.com/article/10.3390/nu17172894/s1. S1: PRISMA 2020 Main Checklist; S2: Bibliographic search; S3: Quality assessment; S4: ROBINS-E.
