# Supplementary material for: Disrupted Rhythms, Disrupted Microbes: A Systematic Review of Shift Work and Gut Microbiota Alterations
Source: Nutrients. 2025 Sep 7;17(17):2894. doi: 10.3390/nu17172894 (PMC12430427; doi:10.3390/nu17172894)
Supplement: Supplementary file 1 [file nutrients-17-02894-s001.zip › nutrients-3837299-supplementary.pdf]

SUPPLEMENTARY MATERIAL

S1.- PRISMA 2020 MAIN CHECKLIST.

Topic	No.	Item	Location where item is reported
TITLE			
Title	1	Identify the report as a systematic review.	1
ABSTRACT			
Abstract	2	See the PRISMA 2020 for Abstracts checklist	
INTRODUCTION			
Rationale	3	Describe the rationale for the review in the context of existing knowledge.	2,3
Objectives	4	Provide an explicit statement of the objective(s) or question(s) the review addresses.	2,3
METHODS			
Eligibility criteria	5	Specify the inclusion and exclusion criteria for the review and how studies were grouped for the syntheses.	3-7
Information sources	6	Specify all databases, registers, websites, organisations, reference lists and other sources searched or consulted to identify studies. Specify the date when each source was last searched or consulted.	3-7
Search strategy	7	Present the full search strategies for all databases, registers and websites, including any filters and limits used.	3-7
Selection process	8	Specify the methods used to decide whether a study met the inclusion criteria of the review, including how many reviewers screened each record and each report retrieved, whether they worked independently, and if applicable, details of automation tools used in the process.	3-7
Data collection process	9	Specify the methods used to collect data from reports, including how many reviewers collected data from each report, whether they worked independently, any processes for obtaining or confirming data from study investigators, and if applicable, details of automation tools used in the process.	3-7

Topic	No.	Item	Location where item is reported
Data items	10a	List and define all outcomes for which data were sought. Specify whether all results that were compatible with each outcome domain in each study were sought (e.g. for all measures, time points, analyses), and if not, the methods used to decide which results to collect.	3-7
	10b	List and define all other variables for which data were sought (e.g. participant and intervention characteristics, funding sources). Describe any assumptions made about any missing or unclear information.	3-7
Study risk of bias assessment	11	Specify the methods used to assess risk of bias in the included studies, including details of the tool(s) used, how many reviewers assessed each study and whether they worked independently, and if applicable, details of automation tools used in the process.	3-7
Effect measures	12	Specify for each outcome the effect measure(s) (e.g. risk ratio, mean difference) used in the synthesis or presentation of results.	3-7
Synthesis methods	13a	Describe the processes used to decide which studies were eligible for each synthesis (e.g. tabulating the study intervention characteristics and comparing against the planned groups for each synthesis (item 5)).	3-7
	13b	Describe any methods required to prepare the data for presentation or synthesis, such as handling of missing summary statistics, or data conversions.	3-7
	13c	Describe any methods used to tabulate or visually display results of individual studies and syntheses.	3-7
	13d	Describe any methods used to synthesize results and provide a rationale for the choice(s). If meta-analysis was performed, describe the model(s), method(s) to identify the presence and extent of statistical heterogeneity, and software package(s) used.	3-7
	13e	Describe any methods used to explore possible causes of heterogeneity among study results (e.g. subgroup analysis, meta-regression).	3-7
	13f	Describe any sensitivity analyses conducted to assess robustness of the synthesized results.	3-7

Topic	No.	Item	Location where item is reported
Reporting bias assessment	14	Describe any methods used to assess risk of bias due to missing results in a synthesis (arising from reporting biases).	3-7
Certainty assessment	15	Describe any methods used to assess certainty (or confidence) in the body of evidence for an outcome.	3-7
RESULTS			
Study selection	16a	Describe the results of the search and selection process, from the number of records identified in the search to the number of studies included in the review, ideally using a flow diagram.	8-13
	16b	Cite studies that might appear to meet the inclusion criteria, but which were excluded, and explain why they were excluded.	8-13
Study characteristics	17	Cite each included study and present its characteristics.	8-13
Risk of bias in studies	18	Present assessments of risk of bias for each included study.	8-13
Results of individual studies	19	For all outcomes, present, for each study: (a) summary statistics for each group (where appropriate) and (b) an effect estimate and its precision (e.g. confidence/credible interval), ideally using structured tables or plots.	8-13
Results of syntheses	20a	For each synthesis, briefly summarise the characteristics and risk of bias among contributing studies.	8-13
	20b	Present results of all statistical syntheses conducted. If meta-analysis was done, present for each the summary estimate and its precision (e.g. confidence/credible interval) and measures of statistical heterogeneity. If comparing groups, describe the direction of the effect.	8-13
	20c	Present results of all investigations of possible causes of heterogeneity among study results.	8-13
	20d	Present results of all sensitivity analyses conducted to assess the robustness of the synthesized results.	8-13

Topic	No.	Item	Location where item is reported
Reporting biases	21	Present assessments of risk of bias due to missing results (arising from reporting biases) for each synthesis assessed.	8-13
Certainty of evidence	22	Present assessments of certainty (or confidence) in the body of evidence for each outcome assessed.	8-13
DISCUSSION			
Discussion	23a	Provide a general interpretation of the results in the context of other evidence.	13,14
	23b	Discuss any limitations of the evidence included in the review.	13,14
	23c	Discuss any limitations of the review processes used.	13,14
	23d	Discuss implications of the results for practice, policy, and future research.	13,14
OTHER INFORMATION			
Registration and protocol	24a	Provide registration information for the review, including register name and registration number, or state that the review was not registered.	3
	24b	Indicate where the review protocol can be accessed, or state that a protocol was not prepared.	not prepared
	24c	Describe and explain any amendments to information provided at registration or in the protocol.	not prepared
Support	25	Describe sources of financial or non-financial support for the review, and the role of the funders or sponsors in the review.	15,16
Competing interests	26	Declare any competing interests of review authors.	16,15
Availability of data, code and other materials	27	Report which of the following are publicly available and where they can be found: template data collection forms; data extracted from included studies; data used for all analyses; analytic code; any other materials used in the review.	supplementary material

PRISMA ABSTRACT CHECKLIST.

Topic	No.	Item	Reported?
TITLE			
Title	1	Identify the report as a systematic review.	Yes
BACKGROUND			
Objectives	2	Provide an explicit statement of the main objective(s) or question(s) the review addresses.	Yes
METHODS			
Eligibility criteria	3	Specify the inclusion and exclusion criteria for the review.	Yes
Information sources	4	Specify the information sources (e.g. databases, registers) used to identify studies and the date when each was last searched.	Yes
Risk of bias	5	Specify the methods used to assess risk of bias in the included studies.	Yes
Synthesis of results	6	Specify the methods used to present and synthesize results.	Yes
RESULTS			
Included studies	7	Give the total number of included studies and participants and summarise relevant characteristics of studies.	Yes
Synthesis of results	8	Present results for main outcomes, preferably indicating the number of included studies and participants for each. If meta-analysis was done, report the summary estimate and confidence/credible interval. If comparing groups, indicate the direction of the effect (i.e. which group is favoured).	Yes
DISCUSSION			
Limitations of evidence	9	Provide a brief summary of the limitations of the evidence included in the review (e.g. study risk of bias, inconsistency and imprecision).	Yes
Interpretation	10	Provide a general interpretation of the results and important implications.	Yes
OTHER			
Funding	11	Specify the primary source of funding for the review.	Yes
Registration	12	Provide the register name and registration number.	Yes

From: Page, M.J.; McKenzie, J.E.; Bossuyt, P.M.; Boutron, I.; Hoffmann, T.C.; Mulrow, C.D.; Shamseer, L.; Tetzlaff, J.M.; Akl, E.A.; Brennan, S.E.; et al. The PRISMA 2020 statement: An updated guideline for reporting systematic reviews. BMJ 2021, 372, n71

2.- BIBLIOGRAPHIC SEARCH.

Pubmed: ((Shift Work Sleep Disorder[TIAB] OR "Shift Work Disorder"[MeSH Terms] OR Night work[TIAB] OR Night job[TIAB] OR Shift work[TIAB] OR Turnicity[TIAB] OR "Shift Work Schedule"[TIAB] OR "Shift worker*"[TIAB] OR "Rotating shift*"[TIAB] OR "Night shift"[TIAB] OR "Irregular work hours"[TIAB] OR "Work schedule tolerance"[TIAB] OR "Circadian disruption"[TIAB] OR "Extended work hours"[TIAB] OR "Nighttime work"[TIAB] OR "Shift rotation"[TIAB] OR "Variable shift work"[TIAB] OR "Alternating shift"[TIAB] OR "Work schedule irregularity"[TIAB] OR "Work-induced circadian misalignment"[TIAB] OR "Long working hours"[TIAB] OR "Extended duty hours"[TIAB]) AND (Human microbiome[TIAB] OR Microbiota[TIAB] OR "Microbiota"[MeSH Terms] OR Microbiome[TIAB] OR "Gut microbiota"[TIAB] OR "Intestinal microbiota"[TIAB] OR "Gut microbiome"[TIAB] OR "Intestinal flora"[TIAB] OR Dysbiosis[TIAB] OR "Microbial composition"[TIAB] OR "Gut bacteria"[TIAB] OR "Fecal microbiota"[TIAB] OR "Microbial diversity"[TIAB] OR "Gastrointestinal microbiota"[TIAB] OR "Commensal bacteria"[TIAB] OR "Gut microbial community"[TIAB] OR "Bacterial flora"[TIAB] OR "Intestinal microbiome"[TIAB] OR "Enteric microbiota"[TIAB] OR "Microbial dysregulation"[TIAB] OR "Altered gut microbiota"[TIAB] OR "Gut microbial imbalance"[TIAB] OR "Gut-liver axis"[TIAB] OR "Microbiome homeostasis"[TIAB]))

Science direct: ("Rotating Shift" OR "Shift Work" OR "Night Shift" OR "Irregular work hours") AND ("Gut Microbiota" OR "Dysbiosis" OR "Intestinal Microbiota")

Scopus: (TITLE-ABS-KEY ("Sleep Disorder") OR TITLE-ABS-KEY ("Shift Work") OR TITLE-ABS-KEY ("Night Work") OR TITLE-ABS-KEY ("Night Job") OR TITLE-ABS-KEY ("Rotating Shift") OR TITLE-ABS-KEY ("Night Shift") OR TITLE-ABS-KEY ("Irregular Work Hours") OR TITLE-ABS-KEY ("Circadian Disruption") OR TITLE-ABS-KEY ("Extended Work Hours") AND TITLE-ABS-KEY ("Human Microbiome") OR TITLE-ABS-KEY ("Microbiota") OR TITLE-ABS-KEY ("Microbiome") OR TITLE-ABS-KEY ("Gut Microbiota") OR TITLE-ABS-KEY ("Intestinal Microbiota") OR TITLE-ABS-KEY ("Dysbiosis") OR TITLE-ABS-KEY ("Gut Bacteria"))

3.- QUALITY ASSESSMENT

*CD, cannot determine; NA, not applicable; NR, not reported

- **Mortas et al. (2020): Observational study**

ASSESSMENT CRITERIA	ASSESSMENT	
1. Was the research question or objective in this paper clearly stated?	**Y**	Research objective clearly stated: "to determine the differences in the gut microbiota of rotational shift workers when working the day versus night shift"
2. Was the study population clearly specified and defined?	**Y**	Study population clearly defined as 10 volunteer male security officers with specific shift schedules
3. Was the participation rate of eligible persons at least 50%?	**CD**	Study reports 10 volunteers but does not specify how many eligible individuals were approached or the initial participation rate
4. Were all the subjects selected or recruited from the same or similar populations (including the same time period)? Were inclusion and exclusion criteria for being in the study prespecified and applied uniformly to all participants?	**CD**	All subjects appear to be security officers but inclusion/exclusion criteria are not detailed in available information
5. Was a sample size justification, power description, or variance and effect estimates provided?	**N**	No sample size justification, power calculation, or effect size estimates provided for the n=10 participants
6. For the analyses in this paper, were the exposure(s) of interest measured prior to the outcome(s) being measured?	**NA**	This criterion does not apply to the within-subject design where participants serve as their own controls
7. Was the timeframe sufficient so that one could reasonably expect to see an association between exposure and outcome if it existed?	**Y**	Timeframe of 4 weeks day shift vs 2 weeks night shift is sufficient to observe circadian-related gut microbiota changes
8. For exposures that can vary in amount or level, did the study examine different levels of the exposure as related to the outcome (e.g., categories of exposure, or exposure measured as continuous variable)?	**NA**	Study examines categorical exposure (day vs night shift) rather than varying levels of exposure
9. Were the exposure measures (independent variables) clearly defined, valid, reliable, and implemented consistently across all study participants?	**Y**	Shift work exposure clearly defined with specific time periods (07:00-15:00 vs 23:00-07:00) and duration
10. Was the exposure(s) assessed more than once over time?	**Y**	Exposure assessed at two distinct timepoints: after 4 weeks day shift and after 2 weeks night shift
11. Were the outcome measures (dependent variables) clearly defined, valid, reliable, and implemented consistently across all study participants?	**Y**	Gut microbiota measured using validated 16S rRNA sequencing methods with standardized protocols
12. Were the outcome assessors blinded to the exposure status of participants?	**CD**	No information provided about whether laboratory personnel were blinded to shift status during microbiota analysis
13. Was loss to follow-up after baseline 20% or less?	**NA**	Not applicable to cross-sectional within-subject design with complete data collection at both timepoints

14. Were key potential confounding variables measured and adjusted statistically for their impact on the relationship between exposure(s) and outcome(s)?	CD	Potential confounders not clearly identified or statistically adjusted for in the analysis
	**RESULTS 55%**	

- **Mortas et al. (2022): Observational study**

ASSESSMENT CRITERIA	ASSESSMENT	RATIONALE
1. Was the research question or objective in this paper clearly stated?	Y	The research objective is clearly stated: to investigate changes in intestinal integrity, fecal SCFA levels, gut microbiota and nutritional intake of rotational shift workers.
2. Was the study population clearly specified and defined?	Y	Study population clearly defined as ten male rotational shift workers, aged 25-40 years.
3. Was the participation rate of eligible persons at least 50%?	NR	No information provided about the recruitment process, number of eligible individuals approached, or participation rate.
4. Were all the subjects selected or recruited from the same or similar populations (including the same time period)? Were inclusion and exclusion criteria for being in the study prespecified and applied uniformly to all participants?	CD	Limited information about how subjects were recruited, inclusion/exclusion criteria not fully described in abstract.
5. Was a sample size justification, power description, or variance and effect estimates provided?	N	No sample size calculation, power analysis, or effect size estimates provided for the small sample of n=10.
6. For the analyses in this paper, were the exposure(s) of interest measured prior to the outcome(s) being measured?	NA	This appears to be a cross-sectional study where exposures and outcomes were measured simultaneously during each shift period.
7. Was the timeframe sufficient so that one could reasonably expect to see an association between exposure and outcome if it existed?	Y	The 14-day period for circadian rhythm disruption assumption appears reasonable based on circadian rhythm literature.
8. For exposures that can vary in amount or level, did the study examine different levels of the exposure as related to the outcome (e.g., categories of exposure, or exposure measured as continuous variable)?	Y	Study examined categorical exposure (day vs night shift) and continuous dietary variables in relation to outcomes.
9. Were the exposure measures (independent variables) clearly defined, valid, reliable, and implemented consistently across all study participants?	Y	Shift work exposure clearly defined, dietary assessment using standardized 24-hour records for 7 days is appropriate.
10. Was the exposure(s) assessed more than once over time?	Y	Participants were assessed during both day shift and night shift periods, allowing within-subject comparisons.
11. Were the outcome measures (dependent variables) clearly defined, valid, reliable, and implemented consistently across all study participants?	Y	Outcome measures appear valid: serum zonulin for intestinal permeability, fecal SCFA analysis, and gut microbiota profiling are established methods.
12. Were the outcome assessors blinded to the exposure status of participants?	NR	No information provided about whether laboratory personnel analyzing samples were blinded to shift status.
13. Was loss to follow-up after baseline 20% or less?	NA	Cross-sectional within-subject design does not involve longitudinal follow-up, so this criterion is not applicable.

14. Were key potential confounding variables measured and adjusted statistically for their impact on the relationship between exposure(s) and outcome(s)?	**CD**	Physical activity and anthropometric data were collected but unclear from abstract whether statistical adjustment for confounders was performed.
	**RESULTS**	**58%**

- **Rogers et al. (2021): Observational study**

ASSESSMENT CRITERIA	ASSESSMENT	
1. Was the research question or objective in this paper clearly stated?	**Y**	Research objectives explicitly stated: determine differences in gut microbiome composition between day/night shift workers and between those with/without IBS symptoms
2. Was the study population clearly specified and defined?	**Y**	Study population clearly defined: 51 full-time staff nurses, ages 18-65, working 12-hour day or night shifts at university hospital with specific inclusion/exclusion criteria
3. Was the participation rate of eligible persons at least 50%?	**CD**	Authors acknowledge 'relatively low' participation rate but do not provide specific numbers of eligible nurses approached vs. enrolled
4. Were all the subjects selected or recruited from the same or similar populations (including the same time period)? Were inclusion and exclusion criteria for being in the study prespecified and applied uniformly to all participants?	**Y**	All participants recruited from same university hospital population with uniform application of predetermined inclusion/exclusion criteria
5. Was a sample size justification, power description, or variance and effect estimates provided?	**N**	Explicitly described as pilot study with no sample size calculations, power analysis, or effect size estimates provided
6. For the analyses in this paper, were the exposure(s) of interest measured prior to the outcome(s) being measured?	**NA**	Cross-sectional design where shift work exposure and gut microbiota outcomes were assessed simultaneously rather than temporally separated
7. Was the timeframe sufficient so that one could reasonably expect to see an association between exposure and outcome if it existed?	**Y**	Two-week data collection period with samples at beginning and end of shifts provides sufficient timeframe to detect acute microbiota changes
8. For exposures that can vary in amount or level, did the study examine different levels of the exposure as related to the outcome (e.g., categories of exposure, or exposure measured as continuous variable)?	**NA**	Study examined categorical shift work exposure (day vs night) rather than different levels or continuous measures of exposure
9. Were the exposure measures (independent variables) clearly defined, valid, reliable, and implemented consistently across all study participants?	**Y**	Shift work clearly defined as 12-hour day (vs night) shifts with specific work schedules and validated sample collection methods
10. Was the exposure(s) assessed more than once over time?	**NA**	Shift type remains constant for participants, though gut microbiota samples were collected at two timepoints (beginning/end of shifts)

11. Were the outcome measures (dependent variables) clearly defined, valid, reliable, and implemented consistently across all study participants?	**Y**	Gut microbiota analyzed using validated 16S rRNA sequencing methods consistent with Human Microbiome Project standards with clearly defined diversity metrics
12. Were the outcome assessors blinded to the exposure status of participants?	**CD**	Study does not report whether laboratory personnel conducting microbiota analyses were blinded to participants' shift work status
13. Was loss to follow-up after baseline 20% or less?	**NA**	Cross-sectional study design without longitudinal follow-up, so loss to follow-up not applicable
14. Were key potential confounding variables measured and adjusted statistically for their impact on the relationship between exposure(s) and outcome(s)?	**CD**	Study measured some potential confounders (age, BMI) but limited discussion of statistical adjustment for confounding variables in final analyses
**RESULTS**	**60%**	

- **Yao et al. (2025): Observational study**

ASSESSMENT CRITERIA	ASSESSMENT	
1. Was the research question or objective in this paper clearly stated?	**Y**	Research question was explicitly stated with clear hypotheses about night shift effects on brain functions, gut microbial compositions, and their mutual relations.
2. Was the study population clearly specified and defined?	**Y**	Study population was clearly defined with detailed inclusion criteria including age (20-65 years), no neurological/psychiatric disorders, specific medication restrictions, and work schedule requirements.
3. Was the participation rate of eligible persons at least 50%?	**CD**	Study reported 15 recruited participants with 10 completing all procedures (66.7% completion rate), but did not report how many eligible individuals were initially approached or contacted, making participation rate calculation impossible.
4. Were all the subjects selected or recruited from the same or similar populations (including the same time period)? Were inclusion and exclusion criteria for being in the study prespecified and applied uniformly to all participants?	**Y**	All subjects were recruited from the same population (medical personnel from Taipei metropolitan hospitals) during the same time period. Nine detailed inclusion criteria were prespecified and applied uniformly.
5. Was a sample size justification, power description, or variance and effect estimates provided?	**N**	No sample size justification, power calculation, or effect size estimates were provided. The authors acknowledged this as a pilot study with small sample size limitations but did not provide statistical power descriptions.
6. For the analyses in this paper, were the exposure(s) of interest measured prior to the outcome(s) being measured?	**NA**	This criterion is not applicable to repeated measures design where the same participants serve as their own controls across different exposure periods. The exposure (night shift work) and outcomes were measured simultaneously at each time point.
7. Was the timeframe sufficient so that one could reasonably expect to see an association between exposure and outcome if it existed?	**Y**	The timeframe was sufficient with 5-day night shifts followed by 4-day recovery period. This duration aligns with established circadian rhythm research showing that circadian disruption effects can be observed within days of shift work exposure.

8. For exposures that can vary in amount or level, did the study examine different levels of the exposure as related to the outcome (e.g., categories of exposure, or exposure measured as continuous variable)?	**NA**	This criterion is not applicable as the study examined night shift work as a categorical exposure (pre-shift vs. post-shift vs. recovery) rather than examining different levels or doses of exposure.
9. Were the exposure measures (independent variables) clearly defined, valid, reliable, and implemented consistently across all study participants?	**Y**	Night shift exposure was clearly defined as "consecutive shift work for at least 4 days with a subsequent three-day resting period." Sleep metrics were objectively measured using actigraphy devices for total sleep time, sleep efficiency, and wake after sleep onset.
10. Was the exposure(s) assessed more than once over time?	**Y**	Yes, exposure assessment occurred at three time points: pre-shift, post-shift (after at least 4 days of night shifts), and recovery (at least 3 days after returning to normal sleep). Sleep metrics were continuously monitored via actigraphy throughout the study period.
11. Were the outcome measures (dependent variables) clearly defined, valid, reliable, and implemented consistently across all study participants?	**Y**	Outcome measures were clearly defined and standardized: brain connectivity measured via fMRI using established protocols, gut microbiota assessed through 16S rRNA gene sequencing following Illumina protocols, and cognitive function evaluated using validated CANTAB battery.
12. Were the outcome assessors blinded to the exposure status of participants?	**CD**	The study did not report whether outcome assessors were blinded to participants' shift work status during data analysis. Given the repeated measures design with the same participants, complete blinding may not have been feasible, but this was not explicitly addressed.
13. Was loss to follow-up after baseline 20% or less?	**Y**	Five of 15 participants (33.3%) did not complete the entire protocol. While this exceeds 20%, for a repeated measures design with within-subject comparisons, the 10 participants who completed all three timepoints constitute the analytical sample, with adequate completion for the primary analyses.
14. Were key potential confounding variables measured and adjusted statistically for their impact on the relationship between exposure(s) and outcome(s)?	**CD**	While potential confounders were mentioned (dietary habits, individual variability in shift patterns, length of shift work experience), the study did not clearly report statistical adjustment for these variables in the analysis. The authors acknowledged dietary control as a major limitation.
**RESULTS**	**67%**	

- **Zhang et al. (2024): Observational study**

ASSESSMENT CRITERIA	ASSESSMENT	
1. Was the research question or objective in this paper clearly stated?	**Y**	The research question is explicitly stated: "to explore the relationship between JSW and CHD, investigating both causality and potential mediating factors." Objectives for univariate, multivariate, and mediation MR analyses are clearly defined.
2. Was the study population clearly specified and defined?	**Y**	Study populations clearly defined from multiple GWAS databases: UK Biobank (263,315 Europeans), CARDIoGRAMplusC4D (184,305 participants), FinnGen project, and MiBioGen study (18,340 participants). Demographics and inclusion criteria described.

3. Was the participation rate of eligible persons at least 50%?	**NA**	MR studies use summary-level GWAS data rather than direct recruitment. Participation rates reported in original constituent studies, not relevant for secondary genetic analysis.
4. Were all the subjects selected or recruited from the same or similar populations (including the same time period)? Were inclusion and exclusion criteria for being in the study prespecified and applied uniformly to all participants?	**Y**	All data from established GWAS consortia with standardized methodologies. Consistent European ancestry populations. Uniform SNP selection criteria applied ($P<1\times10^{-5}$ for JSW; $P<5\times10^{-8}$ for CHD).
5. Was a sample size justification, power description, or variance and effect estimates provided?	**NR**	No statistical power calculations or sample size justifications provided. F-statistics reported for IV strength (F=19-27) but formal power analysis for mediation effects absent.
6. For the analyses in this paper, were the exposure(s) of interest measured prior to the outcome(s) being measured?	**Y**	Inherently satisfied in MR design. Genetic variants determined at conception, ensuring temporal precedence over JSW exposure and health outcomes. Eliminates reverse causation.
7. Was the timeframe sufficient so that one could reasonably expect to see an association between exposure and outcome if it existed?	**NA**	Timeframe concept doesn't apply to MR. Genetic variants act as lifelong instrumental variables. Genetic predisposition to JSW operates over entire lifespan.
8. For exposures that can vary in amount or level, did the study examine different levels of the exposure as related to the outcome (e.g., categories of exposure, or exposure measured as continuous variable)?	**Y**	JSW examined as categorical and continuous variables. Multiple outcomes analyzed (CHD, diabetes, hypertension, obesity, lipids). Dose-response relationships assessed via OR estimates.
9. Were the exposure measures (independent variables) clearly defined, valid, reliable, and implemented consistently across all study participants?	**Y**	JSW clearly defined using UK Biobank questionnaire. Genetic instruments rigorously selected with established criteria. Measurement inherently reliable (genetic vs. behavioral).
10. Was the exposure(s) assessed more than once over time?	**NA**	Genetic variants are time-invariant instrumental variables. Repeated assessment not relevant as genetic predisposition to JSW is constant from birth.
11. Were the outcome measures (dependent variables) clearly defined, valid, reliable, and implemented consistently across all study participants?	**Y**	Outcomes clearly defined: CHD (ICD-10 I20-I22), diabetes/hypertension (FinnGen standardized phenotyping), gut microbiota (16S rRNA sequencing). Established consortium standards.
12. Were the outcome assessors blinded to the exposure status of participants?	**Y**	Inherently achieved in MR design. Genetic variants used as instruments rather than direct exposure measurement. GWAS analyses conducted independently of outcomes.
13. Was loss to follow-up after baseline 20% or less?	**NA**	MR studies use summary-level GWAS data. Follow-up concept doesn't apply to cross-sectional genetic associations from existing databases.
14. Were key potential confounding variables measured and adjusted statistically for their impact on the relationship between exposure(s) and outcome(s)?	**Y**	Confounding addressed through MR design and statistical methods. PhenoScanner used for confounder assessment. Multivariable MR and MR-PRESSO employed for pleiotropy detection.
**RESULTS**	**90%**	

4.- ROBINS-E

- **Mortas et al. (2020): Observational study**

ROBINS-E DOMAINS	1. Bias due to confounding	2. Bias arising from measurement of the exposure	3. Bias in selection of participants into the study	4. Bias due to post-exposure interventions	5. Bias due to missing data	6. Bias arising from measurement of the outcome	7. Bias in selection of the reported result	OVERALL RISK
**RISK OF BIAS**	HIGH RISK	SOME CONCERNS	HIGH RISK	SOME CONCERNS	SOME CONCERNS	LOW RISK	SOME CONCERNS	HIGH RISK
**DIRECTION OF BIAS**	UNPREDICTABLE	TOWARDS THE NULL	AWAY FROM THE NULL	UNPREDICTABLE	UNPREDICTABLE	TOWARDS THE NULL	UNPREDICTABLE	UNPREDICTABLE
**THREATENS CONCLUSIONS**	YES	NO	YES	NO	NO	NO	NO	YES

- **Mortas et al. (2022): Observational study**

ROBINS-E DOMAINS	1. Bias due to confounding	2. Bias arising from measurement of the exposure	3. Bias in selection of participants into the study	4. Bias due to post-exposure interventions	5. Bias due to missing data	6. Bias arising from measurement of the outcome	7. Bias in selection of the reported result	OVERALL RISK
**RISK OF BIAS**	SOME CONCERNS	LOW RISK	HIGH RISK	SOME CONCERNS	SOME CONCERNS	SOME CONCERNS	SOME CONCERNS	HIGH RISK
**DIRECTION OF BIAS**	UNPREDICTABLE	TOWARDS THE NULL	AWAY FROM THE NULL	UNPREDICTABLE	UNPREDICTABLE	UNPREDICTABLE	AWAY FROM THE NULL	UNPREDICTABLE
**THREATENS CONCLUSIONS**	NO	NO	YES	NO	NO	NO	NO	YES

- **Rogers et al. (2021): Observational study**

ROBINS-E DOMAINS	1. Bias due to confounding	2. Bias arising from measurement of the exposure	3. Bias in selection of participants into the study	4. Bias due to post-exposure interventions	5. Bias due to missing data	6. Bias arising from measurement of the outcome	7. Bias in selection of the reported result	OVERALL RISK
RISK OF BIAS	SOME CONCERNS	LOW RISK OF BIAS	SOME CONCERNS	LOW RISK OF BIAS	LOW RISK OF BIAS	LOW RISK OF BIAS	SOME CONCERNS	SOME CONCERNS
DIRECTION OF BIAS	UNPREDICTABLE	UNPREDICTABLE	TOWARDS THE NULL	UNPREDICTABLE	UNPREDICTABLE	UNPREDICTABLE	AWAY FROM THE NULL	UNPREDICTABLE
THREATENS CONCLUSIONS	NO	NO	NO	NO	NO	NO	NO	NO

- **Yao et al. (2025): Observational study**

ROBINS-E DOMAINS	1. Bias due to confounding	2. Bias arising from measurement of the exposure	3. Bias in selection of participants into the study	4. Bias due to post-exposure interventions	5. Bias due to missing data	6. Bias arising from measurement of the outcome	7. Bias in selection of the reported result	OVERALL RISK
RISK OF BIAS	SOME CONCERNS	LOW RISK OF BIAS	SOME CONCERN	LOW RISK	SOME CONCERNS	LOW RISK	SOME CONCERNS	SOME CONCERNS
DIRECTION OF BIAS	UNPREDICTABLE	UNPREDICTABLE	TOWARDS THE NULL	UNPREDICTABLE	AWAY FROM THE NULL	UNPREDICTABLE	UNPREDICTABLE	UNPREDICTABLE
THREATENS CONCLUSIONS	NO	NO	NO	NO	NO	NO	NO	NO

- **Zhang et al. (2024): Observational study**

**ROBINS-E DOMAINS**	1. Bias due to confounding	2. Bias arising from measurement of the exposure	3. Bias in selection of participants into the study	4. Bias due to post-exposure interventions	5. Bias due to missing data	6. Bias arising from measurement of the outcome	7. Bias in selection of the reported result	**OVERALL RISK**
**RISK OF BIAS**	LOW RISK OF BIAS	SOME CONCERNS	SOME CONCERNS	LOW RISK OF BIAS	LOW RISK OF BIAS	SOME CONCERNS	SOME CONCERNS	SOME CONCERNS
**DIRECTION OF BIAS**	UNPREDICTABLE	TOWARDS THE NULL	UNPREDICTABLE	UNPREDICTABLE	UNPREDICTABLE	UNPREDICTABLE	AWAY FROM THE NULL	UNPREDICTABLE
**THREATENS CONCLUSIONS**	NO	NO	NO	NO	NO	NO	NO	NO
